# A downstream box fusion allows stable accumulation of a bacterial cellulase in *Chlamydomonas reinhardtii* chloroplasts

**DOI:** 10.1186/s13068-018-1127-7

**Published:** 2018-05-10

**Authors:** Lubna V. Richter, Huijun Yang, Mohammad Yazdani, Maureen R. Hanson, Beth A. Ahner

**Affiliations:** 1 Department of Biological and Environmental Engineering, Cornell University, 111 Wing Drive, Ithaca, NY USA; 2 Department of Molecular Biology and Genetics, Cornell University, Biotechnology Building, Ithaca, NY USA

**Keywords:** Recombinant protein, Transplastomic, Expression, Plastid, Algae, Cel6A

## Abstract

**Background:**

We investigated strategies to improve foreign protein accumulation in the chloroplasts of the model algae *Chlamydomonas reinhardtii* and tested the outcome in both standard culture conditions as well as one pertinent to algal biofuel production. The downstream box (DB) of the *TetC* or *NPTII* genes, the first 15 codons following the start codon, was *N*-terminally fused to the coding region of *cel6A*, an endoglucanase from *Thermobifida fusca*. We also employed a chimeric regulatory element, consisting of the *16S* rRNA promoter and the *atpA* 5′UTR, previously reported to enhance protein expression, to regulate the expression of the TetC-*cel6A* gene. We further investigated the accumulation of TetC-Cel6A under *N*-deplete growth conditions.

**Results:**

Both of the DB fusions improved intracellular accumulation of Cel6A in transplastomic *C. reinhardtii* strains though the TetC DB was much more effective than the NPTII DB. Furthermore, using the chimeric regulatory element, the TetC-Cel6A protein accumulation displayed a significant increase to 0.3% total soluble protein (TSP), whereas NPTII-Cel6A remained too low to quantify. Comparable levels of TetC- and NPTII-*cel6A* transcripts were observed, which suggests that factors other than transcript abundance mediate the greater TetC-Cel6A accumulation. The TetC-Cel6A accumulation was stable regardless of the growth stage, and the transplastomic strain growth rate was not altered. When transplastomic cells were suspended in *N*-deplete medium, cellular levels of TetC-Cel6A increased over time along with TSP, and were greater than those in cells suspended in *N*-replete medium.

**Conclusions:**

The DB fusion holds great value as a tool to enhance foreign protein accumulation in *C. reinhardtii* chloroplasts and its influence is related to translation or other post-transcriptional processes. Our results also suggest that transplastomic protein production can be compatible with algal biofuel production strategies. Cells displayed a consistent accumulation of recombinant protein throughout the growth phase and nitrogen starvation, a strategy used to induce lipid production in algae, led to higher cellular heterologous protein content. The latter result is contrary to what might have been expected a priori and is an important result for the development of future algal biofuel systems, which will likely require co-products for economic sustainability.

**Electronic supplementary material:**

The online version of this article (10.1186/s13068-018-1127-7) contains supplementary material, which is available to authorized users.

## Background

Photosynthetic microalgae are increasingly being used as a platform for chemical production and may in the future become a viable feedstock for renewable fuel. Well-studied genetically tractable species, such as *Chlamydomonas reinhardtii,* may be used directly for production, e.g. [1] or are used as model systems to develop tools and strategies for ultimate application to other algae species with desired traits. *C. reinhardtii* has been investigated as a host for high-value heterologous protein expression due to its fast reproduction rate, its ability to grow as a heterotroph, and its well-established genetic transformation techniques [2–4]; examples include proteins with value as biopharmaceuticals [5] and in agricultural applications [6].

Chloroplast transformation has several advantages for recombinant protein expression over nuclear transformation. These include precise transgene integration, feasibility to express multiple genes simultaneously, the absence of gene silencing, and the presence of 80–100 identical plastomes per cell resulting in a high transgene copy number [7–9]. Transplastomic *C. reinhardtii* strains do not, however, always accumulate heterologous proteins to detectable levels and strategies to generate strains with consistently high heterologous protein accumulation remain elusive. Many factors can influence plastid transgene expression including host choice [10] and codon usage in the translated region [11]. Modest expression levels have been reported in strains using strong constitutive endogenous promoters (*e.g., rbcL*, *atpA*, *psbA*, *psbD*, and the *rrn* operon) paired with their respective UTRs [3]. The choice of the 3′UTR sequence has been shown to have a minor impact on mRNA stability and protein accumulation [12], whereas the 5′UTR was more influential [12, 13].

The 5′UTR sequence is believed to interact directly with the coding region, forming secondary structures which facilitate the function of translational protein factors. High heterologous protein accumulation (≥ 5% TSP) was achieved when utilizing the endogenous *psbA* promoter and corresponding 5′UTR, but only in a *psbA* knock-out background which is not capable of photosynthesis [14–16]. In photosynthetically competent cells, levels of two proteins, luciferase and 14FN3, were increased when the *16S* rRNA gene promoter and the *atp*A 5′UTR were fused to generate a chimeric regulatory element compared to accumulation in strains in which the endogenous *atpA*, *psbD*, or *rbcL* promoters and 5′ UTRs were used [17]. Fusion of the luciferase gene to the C-terminal of *rbcL*, a highly abundant Rubisco subunit, also increased foreign protein accumulation by three to fivefold relative to use of the *rbcL* promoter and UTRs without the coding region [18].

Slight modification of the *N*-terminus of a transgene can also lead to a dramatic change in plastid expression levels in higher plant chloroplasts [19], but this approach has not been investigated in algae. In tobacco, recombinant protein accumulation improved significantly when the first 10–15 codons, the downstream box (DB), of a highly expressed transgene was fused to the *N*-terminus of another transgene [20]. The downstream box effect is gene dependent such that the same DB sequence can lead to different yields of specific recombinant proteins [20, 21]. Therefore, empirical optimization is needed to determine the appropriate downstream box sequence that will result in high production of a given recombinant protein.

In this study, we investigated two features of the expression cassette and their impact on the production of Cel6A protein in *C. reinhardtii* chloroplasts. The objective of this work was to determine whether the downstream box (DB) would also affect protein accumulation in an algal chloroplast and to evaluate the pairing of potential DB improvements with those of the previously reported chimeric regulatory element on the accumulation of Cel6A. Cel6A is an endoglucanase belonging to a group of commercially important enzymes that hydrolyze cellulose [22], and was chosen for the study because it was successfully and variably expressed in tobacco chloroplasts with DB modifications [20]. We also evaluated the impact of growth conditions used to trigger lipid biosynthesis on foreign protein production, given that one impetus for studying protein expression in algae is to increase the value of protein co-products in algal biofuel production systems.

## Methods

### *Chlamydomonas* strain and growth conditions

*Chlamydomonas reinhardtii* wild-type CC-125 strain and the engineered transformants were maintained in minimal media (20 mM Tris, 0.68 mM K_2_HPO_4_, 7.26 mM KH_2_PO_4_, and 7.5 mM NH_4_Cl) [23], with the addition of 150 µg/mL spectinomycin for the transplastomic strains. Experiments were performed in 50 mL batch cultures of either minimal medium or TAP medium (20 mM Tris, 17 mM Acetate, 0.68 mM K_2_HPO_4_, 7.26 mM KH_2_PO_4_, and 7.5 mM NH_4_Cl) [23], with trace metal additions prepared as described previously [24]. All experimental cultures were incubated at 25 °C with constant shaking (110 rpm), open to the atmosphere and exposed to continuous fluorescent white light (80 µmol/m^2^ s) unless otherwise stated. Cellular growth was monitored by measuring the auto-fluorescence of chlorophyll using a 10-AU Fluorometer (Turner Designs, San Jose, CA), equipped with a Daylight White Lamp F4T5D (EX: 340–500 nm, EM: > 665 nm). Cell counts were made using a Neubauer hemacytometer (Spencer Bright Line^®^) at 40× magnification with an optical microscope (model 1864, Southern Precision Instruments, San Antonio, TX, USA).

### Cloning and plasmid construction

**Fig. 1:**
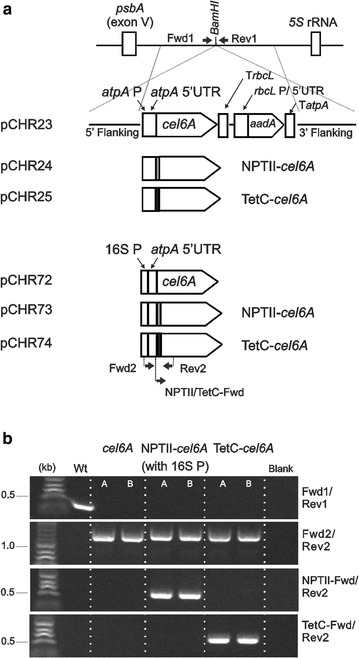
Engineered constructs and site of integration of recombinant genes into the *C. reinhardtii* chloroplast genome. **a** Schematic diagram of the transformation vectors used to integrate the recombinant genes into the inverted repeat of the chloroplast. The *cel6A* coding region is regulated by the endogenous *atpA* promoter, 5′UTR, and terminator in pCHR23, pCHR24, and pCHR25 plasmids; and by the 16S promoter and *atpA* 5′UTR chimeric construct and the *atpA* terminator in pCHR72, pCHR73, and pCHR74 plasmids. The *cel6A* gene is cloned with no added downstream box (DB) in pCHR23 and pCHR72, with the NPTII DB in pCHR24 and pCHR73, and the TetC DB in pCHR25 and pCHR74 plasmids. Primers used to screen for accurate gene insertion and homoplasmicity are indicated by arrows. **b** DNA agarose gels of PCR products generated using the *C. reinhardtii* cell lysate and gene-specific primers. The Fwd1 and Rev1 primers were designed around the insertion site. PCR reaction conditions using the Fwd1 and Rev1 primers were chosen to generate amplicons < 500 bp. Of all the tested strains, only the parent wild-type template gave a product, which confirms the homoplasmic state of the engineered strains (top gel). Fwd2 and Rev2 primers were designed within the *cel6A* sequence and used to confirm transgene integration (second gel from the top). NPTII-Fwd and TetC-Fwd designed within the respective DB sequence and were used in pairs with Rev2 primer to confirm the transgene identity (bottom two gels)

Expression cassettes assembled as illustrated in Fig. 1a were cloned at the *EcoRI* and *SalI* restriction sites of the pUC19 vector. The regulatory elements were amplified from the *C. reinhardtii* chloroplast genome. The chimeric construct of the *16S* rRNA gene promoter and the *atpA* 5′UTR was engineered as described previously [17]. The coding sequence of the *Thermobifida fusca cel6A* (lacking the signal peptide), TetC-*cel6A*, and NPTII-*cel6A* genes were amplified from the pGG86, pTetCCel6A, and the pNPTIICel6A plasmids, respectively [20]. The *aadA* gene, a selectable marker conferring resistance to spectinomycin and streptomycin [25], was amplified from the pPTDNA-aadA plasmid [20]. DNA sequences from a non-coding region between the *psbA* and *5S* rRNA genes were used to flank the expression cassettes (Fig. 1a), which allow integration at the unique *BamHI* site [12] via homologous recombination [26]. DNA fragments were amplified via PCR using a high fidelity Phusion polymerase (Finnzymes, Thermo Fisher Scientific, Waltham, MA), and assembled by restriction digestion and ligation reactions (NEB, Ipswich, MA). Primers used for cloning are listed in Additional file 1: Table S1.

### Chloroplast transformation

*C. reinhardtii* chloroplasts were transformed via particle bombardment with a biolistic device (BioRad, Hercules, CA) [27]. Briefly, *C. reinhardtii* wild-type cells were grown to a density of 1 × 10^6^ cell/mL. Cells were harvested at 2700×*g* for 5 min and resuspended in minimal media to a concentration of 5 × 10^7^ cell/mL. Concentrated cultures were spread in the center (3 cm in diameter) of minimal media plates supplemented with 150 µg/mL spectinomycin, and bombarded with 1-µm gold particles (BioRad, Hercules, CA) coated with the appropriate plasmid DNA. Following bombardment, plates were incubated under dim light until colonies appeared. Several rounds of selection on minimal media with 150 µg/mL spectinomycin were conducted to obtain a homoplasmic cell line.

### PCR screening for transformants

To confirm the accurate transgene placement and to assess the homoplasmy of the engineered transplastomic strains, PCR reactions were performed using sets of primers listed in Additional file 1: Table S1. Wild-type and transplastomic cells were treated with 10 mM Tris–EDTA lysis buffer and boiled for 10 min in preparation for PCR tests [28]. Taq polymerase (NEB, Ipswich, MA) was used in all PCR reactions for 35 cycles following the manufacturer’s instructions. Gene-specific primers were used to confirm the proper transgene insertion. To confirm the homoplasmy of the transformants, Fwd1 and Rev1 primers (Additional file 1: Table S1) were designed to amplify a sequence at the insertion site that is disrupted if the homologous recombination is successful.

### SDS-PAGE and immunoblotting

The wild-type and transplastomic *C. reinhardtii* cultures were grown in TAP or minimal media and harvested by centrifugation (5000×*g* for 15 min) at the growth phase indicated for each experiment. Pellets were resuspended in 2 mL of 10 mM sodium phosphate buffer pH 6.8 with 1 mM PMSF [29]. Resuspended pellets were frozen-thawed for three cycles for a minimum of 1.5 h for each freezing cycle. Cells were then subjected to three rounds of probe sonication with 20/40 s on/off duty cycle on ice. The cell debris was separated from the supernatant by micro-centrifugation at 13,000 rpm for 10 min, and the soluble fraction was stored at − 20 °C until used. The total soluble protein (TSP) was quantified by Bradford assays (BioRad, Hercules, CA).

TSP samples were denatured by the addition of 2× Laemmli SDS-loading buffer (BioRad, Hercules, CA) and boiling for 5 min, then electrophoresed in 12% polyacrylamide pre-cast gels (BioRad, Hercules, CA), and transferred to PVDF membranes (BioRad, Hercules, CA). Membranes were blocked by incubation with 5% (w/v) milk in TBST (0.1 M Tris, pH 7.4, 0.15 M NaCl, 0.1% Tween 20) and then probed with a polyclonal anti-Cel6A primary antibody (kindly donated by David Wilson, Cornell University, Ithaca, NY) for 1 h. The anti-Cel6A primary antibody was diluted 1:10,000 in Antibody Signal Enhancer solution (Amresco, Solon, OH). The secondary antibody, ECL peroxidase-labeled anti-rabbit antibody (GE Healthcare Life Sciences, Marlborough, MA), was diluted 1:20,000 in Antibody Signal Enhancer solution. Membranes were incubated with Clarity Western ECL Substrate solution (BioRad, Hercules, CA) for 1–2 min and visualized on CL-Xposure film (Thermo Fisher Scientific). Exposure time varied depending on the signal intensity. Purified Cel6A protein was provided by David Wilson (Cornell University, Ithaca, NY). Immunoreactive bands were quantified using ImageJ 1.47 software and the Cel6A concentration in the algal extracts was determined by comparison to the relative blot density of known amounts of a purified Cel6A control in the same gel.

### RNA blotting

Total RNA was extracted from *C. reinhardtii* wild-type and transplastomic strains using TriZol reagent according to manufacturer’s instructions (Invitrogen, Carlsbad, CA). RNA concentration was quantified via spectrophotometric absorption at 260 nm, and RNA samples (20 µg per strain) were electrophoresed on a 1.2% agarose formaldehyde gel in MOPS buffer (20 mM MOPS, 5 mM Na Acetate, and 1 mM EDTA, pH 8.0). After electrophoresis, RNA was transferred to a Hybond N+ membrane (Amersham Biosciences, Piscataway, NJ) and UV crosslinked. RNA was detected by hybridization with ^32^P-labeled *cel6A* gene-specific probe prepared by a Random Labeling kit (Ambion, Thermo Fisher Scientific, Waltham, MA). The membrane was exposed to a PhosphorImager screen for detection (Molecular Dynamics, Amersham Biosciences, Piscataway, NJ).

### Nitrogen starvation time course experiment

The TetC-*cel6A*-expressing strain was cultured in minimal media supplemented with 150 µg/mL spectinomycin until late logarithmic phase (3 × 10^6^ cell/mL). Cultures were then centrifuged at 5000×*g* for 15 min, and pellets were resuspended in minimal media with or without added nitrogen (7.5 mM NH_4_Cl). Biological duplicates of each growth medium for three different incubation times (5, 24 or 48 h) were prepared. Cell counts were measured immediately after resuspension (*t* = 0) and then at the end of each incubation. Cell counts were normalized to that measured at *t* = 0 to determine the fold change in cell density. Cultures were collected and treated as described above for foreign protein quantification.

## Results

### Recombinant constructs and chloroplast transformation

Expression cassettes were designed to generate transplastomic *C. reinhardtii* strains that would express the native *T. fusca cel6A* gene with no codon optimization because the chloroplast and bacterial genomes share a similar codon bias and the native sequence was successfully employed for plastid expression in tobacco [20]. In some cassettes, the downstream box (DB) of either the *TetC* (the fragment C from tetanus toxin) or *NPTII* (the neomycin phosphotransferase reporter enzyme) genes was inserted immediately after the start codon of *cel6A* (Fig. 1a). The expression of the *cel6A* variants was regulated by one of two endogenous promoters, the *atpA* promoter or the 16S rRNA promoter (16SP), cloned upstream of the *atpA* 5′UTR sequence (Fig. 1a). Plasmids containing the expression cassettes were transformed into the *C. reinhardtii* chloroplasts by biolistic particle bombardment and the transgenes were integrated at the *BamHI* site within the intergenic region between the *psbA* and *5S* rRNA genes within the *C. reinhardtii* chloroplast genome (Fig. 1a). Transformants were selected on minimal media plates supplemented with spectinomycin, and multiple rounds of streaking were conducted to isolate homoplasmic cell lines. PCR screening was used to verify that the transgene was inserted in the correct location and that the transplastomic strains were homoplasmic (Fig. 1b).

### Quantification of Cel6A accumulation in *C. reinhardtii* transplastomic strains

**Fig. 2:**
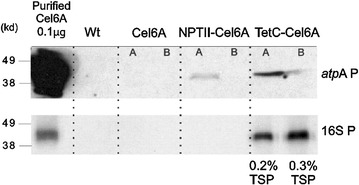
Effect of various regulatory elements on Cel6A accumulation in *C. reinhardtii* transplastomic strains. Immunoblots showing the Cel6A protein accumulation in *C. reinhardtii* strains expressing the *cel6A*, NPTII-*cel6A*, and TetC-*cel6A* genes under the regulation of the *atpA* promoter and 5′UTR (top blot) or the 16S promoter and *atpA* 5′UTR (bottom blot) constructs. Extended exposure time to X-ray film, up to several hours, was used for the top blot, while short exposure time, 1–2 min, was used to observe the immunoreactive bands in the bottom blot. All lanes contained 50 µg total soluble protein (TSP) extracted from the wild-type and transplastomic *C. reinhardtii* strains. Lanes A and B contain protein extracted from two independent transplastomic single colony isolates. Control lane contained 0.1 µg purified Cel6A protein. Quantification of the Cel6A concentration in the algal extracts was done by comparing the relative blot density of bands to those of the purified Cel6A control

Six transplastomic strains engineered to express the *cel6A* gene under the control of different expression element combinations were tested for Cel6A accumulation. When the coding region was prefaced by the *atpA* promoter and 5′UTR, immunoreactive bands of the recombinant protein were visible on blots only after extended exposure time. Notably, TetC-Cel6A and NPTII-Cel6A accumulation was higher than Cel6A but levels were very low and only one transformant of the two tested exhibited detectable protein (Fig. 2, top blot). Examples of different accumulation levels for the same recombinant protein from independent homoplasmic lines have been documented [16].

Previously, the strong endogenous promoter 16SP was reported to boost the plastid expression of the luciferase and 14FN3 genes in *C. reinhardtii* when coupled to the *atpA* 5′UTR, in comparison to the *atpA* promoter and 5′UTR combination [17]. In our experiments, the 16SP and *atpA* 5′UTR chimeric regulatory construct did not improve the native Cel6A or the NPTII-Cel6A production to levels detectable on immunoblots (Fig. 2, bottom blot). However, the chimeric regulatory construct in combination with the TetC DB significantly increased the amount of TetC-Cel6A protein (to ~ 0.3% of total soluble protein, TSP) in both homoplasmic lines (Fig. 2). This result is consistent with earlier work which showed that TetC-Cel6A accumulation was higher than that of NPTII-Cel6A in tobacco [20]. Crude cell extracts of TetC-Cel6A-containing algae were tested for enzymatic activity using Carboxymethyl Cellulose (CMC) as a substrate and showed greater activity than wild-type cell extracts demonstrating that transgenic protein was active (Additional file 2: Figure S1).

### Characterization of *cel6A* mRNA in *C. reinhardtii* transplastomic strains

**Fig. 3:**
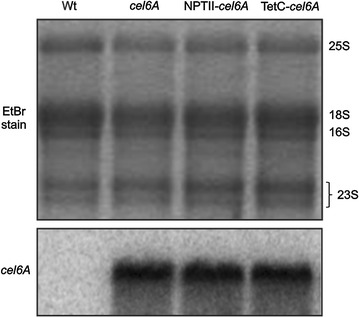
RNA blotting of the wild-type and transplastomic *C. reinhardtii* strains containing the *cel6A* genes regulated by the 16S promoter and *atpA* 5′UTR chimeric construct. The RNA bands from the ethidium-bromide-stained agarose gel are shown in the top gel. Total RNA was hybridized with a radiolabeled *cel6*A probe to evaluate the accumulation of *cel6A* transcripts in *cel6A*, NPTII-*cel6A*, and TetC-*cel6A* transplastomic strains as well as its absence in the wild-type RNA sample (bottom gel)

To evaluate the *cel6A* mRNA expression level when regulated by the 16SP and *atpA* 5′UTR chimeric construct and to investigate a possible correlation between the protein yield observed in Fig. 2 and the transcription of the corresponding transgene, RNA blots were performed (Fig. 3). The stained gel provides visual confirmation that equal amounts of RNA were loaded in each lane (Fig. 3, top gel). Hybridization with a *cel6A*-specific probe revealed similar accumulation of *cel6A* mRNA in all of the transplastomic strains at the predicted size (1.3 kb) (Fig. 3, bottom gel). Consistent with this result, RT-qPCR analysis showed comparable expression levels of the *cel6A*, NPTII-*cel6A,* and TetC-*cel6A* genes when controlled by the 16SP and *atpA* 5′UTR in transplastomic cell-wall mutant strains (Additional file 3: Figure S2). Taken together, the differences in Cel6A accumulation observed on protein blots (Fig. 2, bottom gel and Additional file 3: Figure S2b) are not due to differences in transcript accumulation between strains.

### Transplastomic strain growth and recombinant protein accumulation

**Fig. 4:**
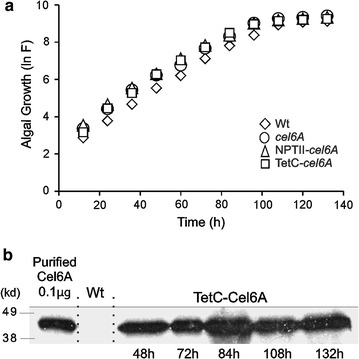
TetC-Cel6A accumulation during growth of transplastomic *C. reinhardtii* strains. **a** Growth curve of *C. reinhardtii* wild-type and transformed strains harboring the *cel6A*, NPTII-*cel6A*, and TetC-*cel6A* genes regulated by the 16SP and *atpA* 5′UTR, grown in TAP media. A representative of triplicate growth curves for each cell line is shown. **b** Evaluation of the TetC-Cel6A protein accumulation throughout the cellular growth phases. The wild-type cell line was used as a negative control. All lanes contained 50 µg total soluble protein (TSP) extracted from either the wild-type or the TetC-*cel6A* strain

To further characterize the strains expressing the DB-*cel6A* variants, growth of the transplastomic strains expressing the *cel6A*, NPTII-*cel6A*, or TetC-*cel6A* under the control of the 16SP and *atpA* 5′UTR chimeric construct, in addition to the wild-type strain, was monitored. Algal growth in TAP medium was quantified by measuring the auto-fluorescence of chlorophyll. All transplastomic strains displayed an average growth rate of 1.75 ± 0.08/day, which was indistinguishable from wild type, and reached a similar maximum cell density (Fig. 4a). Assessment of recombinant TetC-Cel6A protein accumulation throughout the entire cellular growth phase including after 2 days of senescence reveals fairly constant levels, with a modest peak during late exponential phase (*t* = 84 h, Fig. 4b).

### Effect of nitrogen starvation on transplastomic Cel6A accumulation

**Fig. 5:**
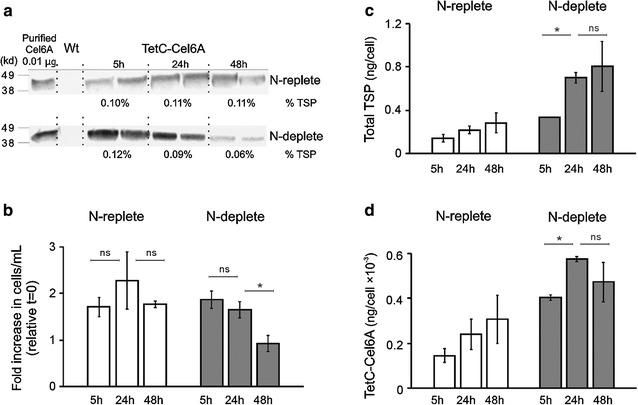
Effect of nitrogen starvation on the accumulation of TetC-Cel6A. **a** Immunoblots showing the TetC-Cel6A protein accumulation after 5, 24, and 48 h of resuspension in *N*-replete (top blot) or *N*-deplete (bottom blot) minimal media. Exposure time was 2 min for both blots. All lanes contained 10 µg total soluble protein. Control lane contained 0.01 µg purified Cel6A protein. The TetC-Cel6A protein accumulation was quantified in two biological replicates by comparing the relative blot density of bands to those of a purified Cel6A control. **b**–**d** Several parameters monitored in the TetC-Cel6A expressing cells grown under N-replete or N-deplete media for 5, 24, and 48 h. Data are the average of two biological replicates per treatment and time point. Error bars are the standard error of the mean. Statistical analysis were performed by *Student’s t test*, *p < 0.05 and ns = not significant

Algae are a potential feedstock for biofuel production and nitrogen deprivation promotes starch and triacylglycerol (TAG) biosynthesis [30]. *N*-starvation also triggers other changes in cell physiology, including the down regulation of most genes involved in photosynthesis and a transition to gametogenesis in some species, including *C. reinhardtii* [31]. To determine the effect of nitrogen starvation on the accumulation of recombinant TetC-Cel6A, cells expressing the TetC-*cel6A* gene regulated by the 16SP and *atpA* 5′UTR chimeric construct were cultivated in minimal media until late logarithmic growth, then pelleted, and resuspended in *N*-free or *N*-replete minimal media for 5, 24, and 48 h. The TetC-Cel6A accumulation was evaluated at each time point using immunoblots (Fig. 5a).

Following resuspension, the transplastomic strain maintained cell division at a similar rate in both media for the first 5 h (fold change in cell counts from *t* = 0 is shown in Fig. 5b). After 48 h, cell numbers were significantly lower in the *N*-deplete cultures, presumably because of gametogenesis triggered by *N*-limitation; similar observations were reported previously [1]. In contrast, the plateau in cell density in the *N*-replete medium was likely caused by carbon limitation or shading.

Distinct changes in cellular protein content, both TSP and TetC-Cel6A, were observed in the two media. After just 5 h, *N*-deplete cells had twice the TSP compared to *N*-replete cells (Fig. 5c), perhaps due to changes in cellular metabolism causing a shift of *N* resources from storage into protein synthesis. Subsequently, the protein content doubled in *N*-deplete cells and then leveled off after 24 and 48 h, respectively, whereas *N*-replete cells did not exhibit significant changes in protein accumulation through the same time period (Fig. 5c). The TetC-Cel6A content of cells (ng/cell) mirrored extractable protein to a large extent, increasing gradually in *N*-replete cells and increasing more rapidly in *N*-deplete cells, with the highest cellular levels recorded after 24 h of *N*-starvation (Fig. 5d). We do not believe that changes in cellular protein content are due to differences in extraction efficiency; at least one other study noted no change in this parameter even as cell wall thickness increased with *N*-depletion [32].

## Discussion

The development of economical and renewable sources of fuel is critical to a sustainable future. One key to the economic success of algae-derived biofuel is the development of protein co-products that may also be of value for biofuel production or sold as animal feed [33, 34]. In this work, we developed transplastomic *C. reinhardtii* strains that can produce Cel6A, an endoglucanase from *T. fusca,* which has been previously shown to accumulate variably in tobacco chloroplasts. The high conservation of chloroplasts across microalgae species makes it likely that regulatory strategies successful in *C. reinhardtii* will be transferrable to related algae with better lipid accumulation traits, such as *Chlorella* C596 [35].

It was previously shown that the expression of two heterologous proteins could be enhanced when the strong *16S* rRNA gene promoter was used with the *atpA* 5′UTR in place of the endogenous *atpA* promoter [17]. In this study, the 16SP and *atp*A 5′UTR chimeric regulatory construct improved protein accumulation in only one of the three engineered constructs, the one in which the TetC downstream box (DB) was *N*-terminally fused to the *cel6A* gene immediately after the start codon (Fig. 2). In the strain containing this construct, TetC-Cel6A accumulated to 0.3% TSP. In tobacco, the DB of both the *TetC* and *NPTII* genes were utilized to enhance plastid production of *cel6A*, resulting in 10.7 and 0.9% TSP for the TetC-Cel6A and NPTII-Cel6A proteins, respectively, compared to 0.1% when using yet another unique DB from GFP [20]. In the tobacco study, the DB sequence was postulated to increase the stability of the mRNA transcript. We have now shown that the TetC DB is more effective than the NPTII DB in conjunction with the *cel*6*A* ORF in *C. reinhardtii* chloroplasts as well (Fig. 2).

Protein accumulation from chloroplast transgenes is likely influenced by several factors including transcription and translation regulators, RNA turnover, and/or protein turnover. Analyses of RNA blots included herein revealed comparable monocistronic *cel6A* transcript accumulation regardless of the presence or identity of the DB (Fig. 3), which leads us to conclude factors other than transcript abundance are mediating recombinant Cel6A accumulation. Coragliotti et al. also showed that changes in mRNA levels of plastid-expressed green fluorescent protein (GFP) and bacterial luciferase did not result in concomitant changes in protein accumulation [36]. They concluded that the main factor limiting recombinant protein accumulation in their study was poor mRNA translation. The authors proposed that the coding region of the transgene might interact with the 5′UTR forming a stable and defined secondary structure which in turn facilitates binding to translation activators [12, 36]. As noted above, the TetC DB improved Cel6A accumulation in both tobacco [20] and in *C. reinhardtii* (Fig. 2); however, the 5′UTRs utilized in these constructs were different. The T7g10 and *atpA* 5′UTR sequences were used in tobacco and *C. reinhardtii,* respectively. Therefore, it is plausible to speculate that the TetC DB along with the *cel6A* coding sequence is actually the critical region for stabilization or successful initiation of translation. This assertion is further supported by the earlier finding in tobacco that while using the same T7g10 5′UTR, the NPTII DB was more effective than the TetC DB when coupled to the coding region of *bglC* [21]. Only when the mechanism whereby the DB enhances the production of recombinant proteins is known will we be able to design a priori an effective DB for a given gene.

With respect to the longer term goal of using algae to produce proteins commercially as a co-product in biofuel production, we have shown that this expression cassette is largely compatible with that goal. In particular, the 16SP and *atpA* 5′UTR regulation resulted in stable TetC-Cel6A accumulation at different growth stages (Fig. 4b) making it possible to grow such algae in continuous or batch cultures with less attention to precise timing of protein harvest. Braun-Galleani et al. reported that cultivation conditions should be optimized for different recombinant proteins, as conditions leading to high accumulation of a given protein would not necessarily be optimal for another [10]. We did observe that TetC-Cel6A accumulation was roughly 60% lower when using photoautotrophic cultivation (0.12% TSP in minimal media) compared to mixotrophic growth (0.3% TSP in TAP media) but we did not attempt any optimization with respect to medium composition.

In production systems, nitrogen starvation is commonly used to induce lipid production. *C. reinhardtii* begins to accumulate starch after 1 day of *N*-starvation and then TAGs after 2–3 more days [37]. During this same interval, vegetative *C. reinhardtii* cells also differentiate into haploid gametes (mt+ and mt−), to prepare for sexual reproduction [38]. Our experiments reveal that accumulation of foreign protein is not compromised when cells are initially shifted to *N*-limitation; in fact during the first 24 h of *N*-limitation, recombinant TetC-Cel6A accumulation is double that measured in *N*-replete cells (Fig. 5d). Numerous physiological transitions occur during *N*-depletion and therefore active protein production machinery is likely required in the chloroplasts. We do not know whether the rapid increase in cellular Cel6A levels is due to a particular regulatory element in our construct or is simply due to a general upregulation of all protein synthesis.

## Conclusions

Our work demonstrates that a foreign downstream box (DB) fusion can significantly enhance the accumulation of a heterologous endoglucanase in *C. reinhardtii* chloroplasts, but a better understanding of the post-transcriptional mechanism involved is still needed to determine how best to increase accumulation of other proteins. We show that when the chimeric regulatory construct of 16SP and 5′UTR was used, the TetC-Cel6A production was unaltered by the cellular growth, though was influenced by medium composition and *N*-limitation. Perhaps contrary to expectations, our experiment reveals a substantial increase in cellular foreign protein accumulation associated with a short period of *N*-deprivation. Further work is needed to determine if this benefit can be realized in conjunction with systems designed to generate lipid-rich biomass. In summary, the downstream box used in conjunction with the right regulatory elements holds great promise for production of recombinant protein production in green algal chloroplasts.

## Additional files

**Additional file 1: Table S1.** Primers used in this work.

**Additional file 2: Figure S1.** The Cel6A enzymatic activities were determined by the Carboxymethyl Cellulose (CMC) assay. Total proteins from wild-type and TetC-*cel6A* cells (50 µg/sample) were incubated with 0.4 M CMC in a water bath at 50 °C for 1 or 2 h. Reactions were quenched by the addition of DNS buffer, and samples were then heated to 95 °C for 10 min for color development. The optical density was assayed at 540 nm to quantify cellobiose accumulation. Sixty minute assays with purified Cel6A were included as a positive control. The wild-type extracts exhibited a baseline enzymatic activity which could be attributed to one or more endogenous enzymes known to be present in *Chlamydomonas reinhardtii*, as previously published by Blifernez-Klassen et al. (2012) [1].

**Additional file 3: Figure S2.** **a** RT-qPCR analyses of *cel6A* expression levels in the chloroplast transformed cell-wall mutants and the wild-type strains. The same cloning strategy described in Fig. 1a (pCHR72, pCHR73, pCHR74) was carried out to generate the *cel6A*, NPTII-*cel6A,* and TetC-*cel6A* cell lines in the cell-wall mutant background (CR4349), via the glass bead method as previously described [2, 3]. The expression of the *cel6*A gene in the transgenic strains is under the regulation of the 16S promoter and *atpA* 5’UTR construct. The transcript levels are shown as a fold-change relative to the expression level of *rbc*L gene (*n* = 3 ± SD; *: *p *> 0.0001). **b** Immunoblot comparison of the Cel6A protein accumulation in the transformed cell-wall mutant strains expressing the *cel6A*, NPTII-*cel6A*, and TetC-*cel6A* genes under the regulation of the 16S promoter and *atpA* 5’UTR construct, and grown in minimal media. Each lane contains 50 µg total soluble protein.

## Abbreviations

DB: downstream box
16SP: *16S* rRNA gene promoter
5′UTR: 5′ untranslated region
3′UTR: 3′ untranslated region
NPTII: the neomycin phosphotransferase reporter enzyme
TetC: the fragment C from tetanus toxin gene
T7g10: gene 10 of the bacteriophage T7
Cel6A: an endoglucanase
*aadA*: aminoglycoside adenine transferase
*atpA*: ATP synthase subunit A
*rbcL*: Rubisco large subunit
TSP: total soluble protein
PCR: polymerase chain reaction
