# Correction to: A downstream box fusion allows stable accumulation of a bacterial cellulase in *Chlamydomonas reinhardtii* chloroplasts

**DOI:** 10.1186/s13068-019-1622-5

**Published:** 2019-12-10

**Authors:** Lubna V. Richter, Huijun Yang, Mohammad Yazdani, Maureen R. Hanson, Beth A. Ahner

**Affiliations:** 1 Department of Biological and Environmental Engineering, Cornell University, 111 Wing Drive, Ithaca, NY USA; 2 Department of Molecular Biology and Genetics, Cornell University, Biotechnology Building, Ithaca, NY USA

## Correction to: Biotechnol Biofuels (2018) 11:133 10.1186/s13068-018-1127-7

In the original version of the article [1], a calculation error resulted in a 3-order of magnitude mistake for the y-axis of the data reported in Fig. 5c and d. The new figures are labeled as “pg/cell” (Fig. 5c, formerly “ng/cell”) and “fg/cell” (Fig. 5d, formerly “ng/cell × 10^−3^”).

**Fig. 5:**
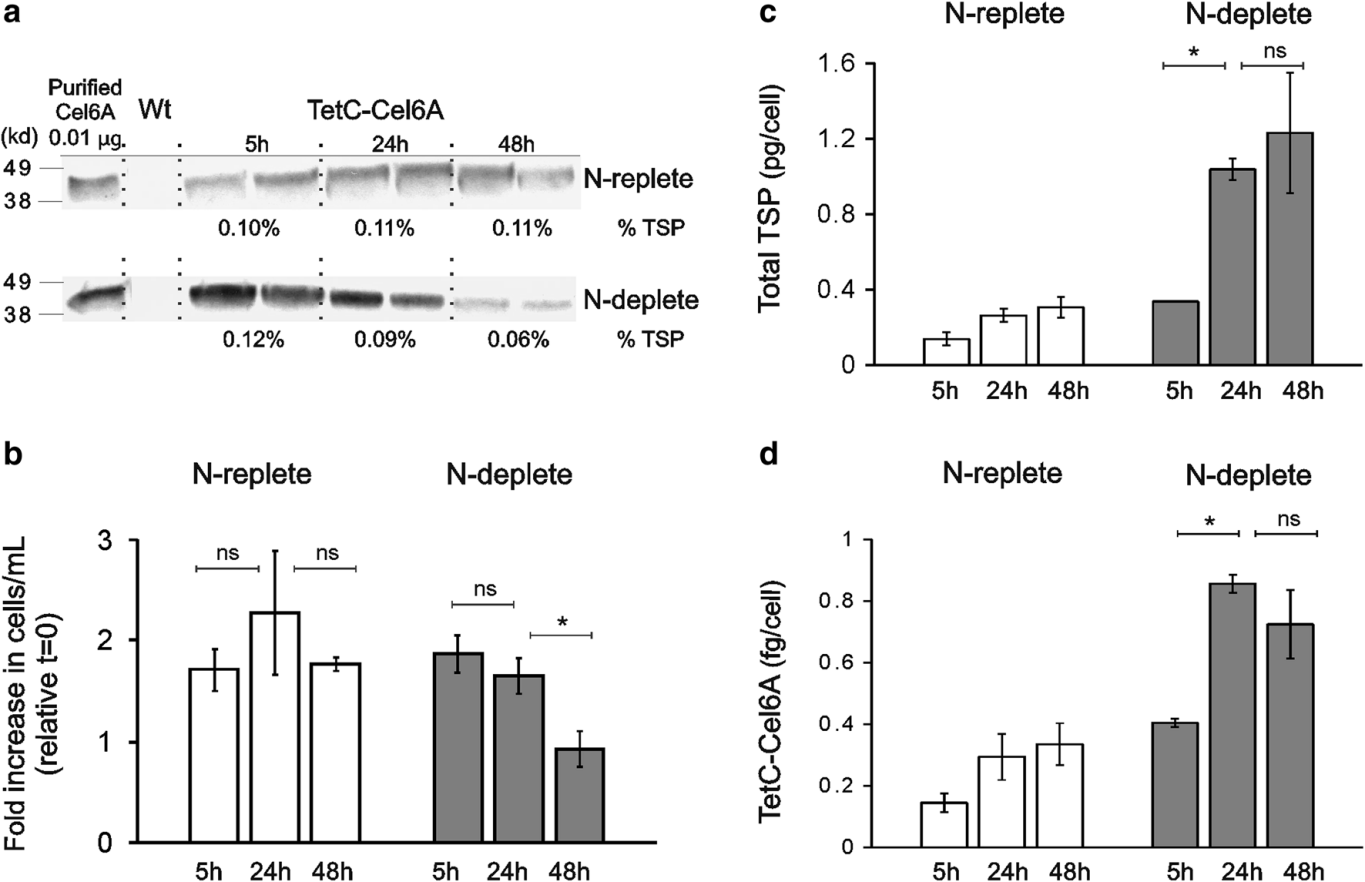
Effect of nitrogen starvation on the accumulation of TetC-Cel6A. **a** Immunoblots showing the TetC-Cel6A protein accumulation after 5, 24, and 48 h of resuspension in *N*-replete (top blot) or *N*-deplete (bottom blot) minimal media. Exposure time was 2 min for both blots. All lanes contained 10 μg total soluble protein. Control lane contained 0.01 μg purified Cel6A protein. The TetC-Cel6A protein accumulation was quantified in two biological replicates by comparing the relative blot density of bands to those of a purified Cel6A control. **b**–**d** Several parameters monitored in the TetC-Cel6A expressing cells grown under *N*-replete or *N*-deplete media for 5, 24, and 48 h. Data are the average of two biological replicates per treatment and time point. Error bars are the standard error of the mean. Statistical analysis were performed by *Student’s t test*, *p < 0.05 and ns = not significant

The only textual reference to these units is on page 6 of the MS. The correct sentence should be:

“The TetC-Cel6A content of cells (pg/cell) mirrored extractable protein to a large extent, increasing gradually in *N*-replete cells and increasing more rapidly in *N*-deplete cells, with the highest cellular levels recorded after 24 h of *N*-starvation (Fig. 5d).”

The correct figure is published in this correction article.
