# Supplementary material for: A downstream box fusion allows stable accumulation of a bacterial cellulase in Chlamydomonas reinhardtii chloroplasts
Source: Biotechnol Biofuels. 2018 May 10;11:133. doi: 10.1186/s13068-018-1127-7 (PMC5944112; doi:10.1186/s13068-018-1127-7)
Supplement: Supplementary file 1 — Additional file 1: Table S1. Primers used in this work. [file 13068_2018_1127_MOESM1_ESM.docx]

**Additional Information**

**A Downstream Box Fusion Allows Stable Accumulation of a Bacterial Cellulase in *Chlamydomonas* *reinhardtii* Chloroplasts**

Lubna V. Richter^1^, Huijun Yang^1, 2^, Mohammad Yazdani^1^, Maureen R. Hanson^2^, and Beth A. Ahner^1*^

^1^ Department of Biological and Environmental Engineering, 111 Wing Drive, Cornell University, Ithaca, New York, USA

^2^ Department of Molecular Biology and Genetics, 321 Biotechnology Building, Cornell University, Ithaca, New York, USA

^*^Correspondence to: B. A. Ahner

[baa7@cornell.edu](mailto:baa7@cornell.edu)

Tel: 607- 255- 4677

Fax: 607-255- 3679

**Table S1**. Primers used in this work

| Name | Sequence 5’- 3’ | Purpose |
| --- | --- | --- |
| Fwd2 | GGC AGG CAA CAA ATT TAT TTA TTG TC | Forward and reverse primers specific for the *cel6*A, NPTII-*cel6*A and TetC-*cel6*A genes to screen for accurate *cel6*A integration. |
| TetC-Fwd | CAT ATG GCT AGC AAA AAT CTG GAT TGT TG |  |
| NPTII-Fwd | CAT ATG GCT AGC ATT GAA CAA GAT GGA TTG |  |
| Rev2 | CAC AGG AAG GCG TCG ATC ATC |  |
| Fwd1 | CAA TTG GAA TAA TTG GAA TTG GAT ATG | Forward and reverse primers specific to the sequence at the insertion site to screen for homoplasmic transformation. |
| Rev1 | GCG ACA GGT ACT TCC GAA ACG GTG |  |
| rHY1 | GTC GAC TGT CGT CTA CGA TAT GTA AAT C | For amplifying the intergenic region between the *psb*A and 5S rRNA genes. *SalI* and *EcoRI* sites are underlined. |
| rHY2 | GAA TTC AAG GGG AAG GAG GAA GCA GGC AG |  |
| rHY3 | GGA TCC GGC AGG CAA CAA ATT TAT TTA TTG TC | For amplifying the 16S rRNA promoter. *BamHI* and *HindIII* sites are underlined. |
| rHY4 | AAG CTT ACT CTT TAA AGT TTA AAT TTT GTC |  |
| rHY5 | GGA TCC TTA GGT ATA TGT CGG TCG TCT TAC G | For amplifying the *atp*A promoter and 5’UTR. *BamHI* and *NdeI* sites are underlined. |
| rHY6 | CAT ATG AAA AAA GAA AAA ATA AAT AAA AGA TTA AAA A |  |
| rHY7 | AAG CTT TTT ACC TTT TTT TTA ATT TGC ATG ATT TTA ATG | For amplifying the *atp*A 5’UTR sequence. *HindIII* and *NdeI* sites are underlined. |
| rHY6 | CAT ATG AAA AAA GAA AAA ATA AAT AAA AGA TTA AAA A |  |
| *cel6*AFwd | CAT ATG *GCT AGC* AAT GAT TCT CCG TTC TAC | For amplifying the *cel6*A gene with no downstream box, and with NPTII or TetC downstream box. *NdeI* site is underlined. *NheI* and *StuI* sites are italicized. |
| NPTII-*cel6*AFwd | CAT ATG *GCT AGC* ATT GAA CAA GAT GGA TTG CAC GCA GGT TCT CCG GCC GCT AAT GAT TCT CCG TTC TAC |  |
| TetC-*cel6*AFwd | CAT ATG *GCT AGC* AAA AAT CTG GAT TGT TGG GTC GAC AAT GAA GAA GAT ATA AAT GAT TCT CCG TTC TAC |  |
| *cel6*ARev | *AGG CCT* TCA GCT GGC GGC GCA GGT AAG |  |
| rHY9 | AGG CCT TTT TAA TTA AGT AGG AAC TCG GTA TAT GC | For amplifying the *atp*A terminator sequence. *StuI* and *BamHI* sites are underlined. |
| rHY10 | GGA TCC TCG TAG AGA TCC TAC GTT TTT TTA GG |  |
| rHY11 | GGA TCC AGT CTA CTA TAT TGG AGA GGA GT | For amplifying the *rbc*L promoter and 5’UTR sequence. *BamHI* and *NdeI* sites are underlined. |
| rHY12 | CAT ATG TTA TAT AAA TAA ATG TAA CTT C |  |
| rHY13 | CAT ATG GCT CGT GAA GCG GTT ATC | For amplifying the *aad*A gene. *NdeI* and *StuI* sites are underlined. |
| rHY14 | AGG CCT TTA TTT GCC GAC TAC CTT GGT GAT C |  |
| rHY15 | AGG CCT TTT TTA TTT TTC ATG ATG TTT ATG TG | For amplifying the *rbc*L terminator sequence. *StuI* and *BamHI* sites are underlined. |
| rHY16 | GGA TCC CAT AAA GAT AAA TTC TAT AAT AAA AAG |  |
| MY_Fwd1 | CGT AGC TTA CCC AAT CGA CTT AT | RT-qPCR primers. For amplifying the *rbc*L gene. |
| MY_Rev1 | TCA AGA CGT AGA GCA CGT AAA G |  |
| MY_Fwd2 | CCA CCA ACA CCT CCA ACT AC | RT-qPCR primers. For amplifying the *cel6*A gene. |
| MY_Rev2 | GTC GCA CCA CTC GTT ACC |  |
