# Supplementary material for: A downstream box fusion allows stable accumulation of a bacterial cellulase in Chlamydomonas reinhardtii chloroplasts
Source: Biotechnol Biofuels. 2018 May 10;11:133. doi: 10.1186/s13068-018-1127-7 (PMC5944112; doi:10.1186/s13068-018-1127-7)
Supplement: Supplementary file 2 — Additional file 2: Figure S1. The Cel6A enzymatic activities were determined by the Carboxymethyl Cellulose (CMC) assay. Total proteins from wild-type and TetC-cel6A cells (50 µg/sample) were incubated with 0.4 M CMC in a water bath at 50 °C for 1 or 2 h. Reactions were quenched by the addition of DNS buffer, and samples were then heated to 95 °C for 10 min for color development. The optical density was assayed at 540 nm to quantify cellobiose accumulation. Sixty minute assays with purified Cel6A were included as a positive control. The wild-type extracts exhibited a baseline enzymatic activity which could be attributed to one or more endogenous enzymes known to be present in Chlamydomonas reinhardtii, as previously published by Blifernez-Klassen et al. (2012) [1]. [file 13068_2018_1127_MOESM2_ESM.docx]

Figure S1

**Fig. S1.**  The Cel6A enzymatic activities were determined by the Carboxymethyl Cellulose (CMC) assay. Total proteins from wild-type and TetC-*cel6A* cells (50 µg/sample) were incubated with 0.4 M CMC in a water bath at 50°C for 1 or 2 hrs. Reactions were quenched by the addition of DNS buffer, and samples were then heated to 95°C for 10 min for color development. The optical density was assayed at 540 nm to quantify cellobiose accumulation. Sixty minute assays with purified Cel6A were included as a positive control. The wild-type extracts exhibited a baseline enzymatic activity which could be attributed to one or more endogenous enzymes known to be present in *Chlamydomonas reinhardtii*, as previously published by Blifernez-Klassen *et al.,* (2012) [1].

1. Blifernez-Klassen O, Klassen V, Doebbe A, Kersting K, Grimm P, Wobbe L, Kruse O: Cellulose degradation and assimilation by the unicellular phototrophic eukaryote *Chlamydomonas reinhardtii*. Nature communications. 2012;3:1214-23.

2. Barrera D, Gimpel J, Mayfield S: Rapid screening for the robust expression of recombinant proteins in algal plastids. Methods Mol Biol. 2014;1132:391-9.

3. Kindle KL: High-frequency nuclear transformation of Chlamydomonas reinhardtii. Proc Natl Acad Sci U S A. 1990;87(3):1228-32.
