# Supplementary material for: A downstream box fusion allows stable accumulation of a bacterial cellulase in Chlamydomonas reinhardtii chloroplasts
Source: Biotechnol Biofuels. 2018 May 10;11:133. doi: 10.1186/s13068-018-1127-7 (PMC5944112; doi:10.1186/s13068-018-1127-7)
Supplement: Supplementary file 3 — Additional file 3: Figure S2. a RT-qPCR analyses of cel6A expression levels in the chloroplast transformed cell-wall mutants and the wild-type strains. The same cloning strategy described in Fig. 1a (pCHR72, pCHR73, pCHR74) was carried out to generate the cel6A, NPTII-cel6A, and TetC-cel6A cell lines in the cell-wall mutant background (CR4349), via the glass bead method as previously described [2, 3]. The expression of the cel6A gene in the transgenic strains is under the regulation of the 16S promoter and atpA 5’UTR construct. The transcript levels are shown as a fold-change relative to the expression level of rbcL gene (n = 3 ± SD; *: p > 0.0001). b Immunoblot comparison of the Cel6A protein accumulation in the transformed cell-wall mutant strains expressing the cel6A, NPTII-cel6A, and TetC-cel6A genes under the regulation of the 16S promoter and atpA 5’UTR construct, and grown in minimal media. Each lane contains 50 µg total soluble protein. [file 13068_2018_1127_MOESM3_ESM.docx]

Figure S2

**Fig. S2. a** RT-qPCR analyses of *cel6A* expression levels in the chloroplast transformed cell-wall mutants and the wild-type strains. The same cloning strategy described in Fig. 1a (pCHR72, pCHR73, pCHR74) was carried out to generate the *cel6A*, NPTII-*cel6A* and TetC-*cel6A* cell lines in the cell-wall mutant background (CR4349), *via* the glass bead method as previously described [2, 3]. The expression of the *cel6*A gene in the transgenic strains is under the regulation of the 16S promoter and *atpA* 5’UTR construct. The transcript levels are shown as a fold‑change relative to the expression level of *rbc*L gene (n= 3 ± SD; *: *p*> 0.0001). **b** Immunoblot comparison of the Cel6A protein accumulation in the transformed cell-wall mutant strains expressing the *cel6A*, NPTII-*cel6A* and TetC-*cel6A* genes under the regulation of the 16S promoter and *atpA* 5’UTR construct, and grown in Minimal media. Each lane contains 50 µg total soluble protein.

1. Blifernez-Klassen O, Klassen V, Doebbe A, Kersting K, Grimm P, Wobbe L, Kruse O: Cellulose degradation and assimilation by the unicellular phototrophic eukaryote *Chlamydomonas reinhardtii*. Nature communications. 2012;3:1214-23.

2. Barrera D, Gimpel J, Mayfield S: Rapid screening for the robust expression of recombinant proteins in algal plastids. Methods Mol Biol. 2014;1132:391-9.

3. Kindle KL: High-frequency nuclear transformation of Chlamydomonas reinhardtii. Proc Natl Acad Sci U S A. 1990;87(3):1228-32.
